# Parkinson’s Disease Bradykinesia, Forward Posture, and Drug-Induced Pisa Syndrome Alleviated With Traditional Japanese Acupuncture: A Case Report

**DOI:** 10.7759/cureus.70860

**Published:** 2024-10-04

**Authors:** Takuya Masuda, Kenichiro Egawa, Yu Takeshita, Koichiro Tanaka

**Affiliations:** 1 Division of General Internal Medicine & Rheumatology, Mitsui Memorial Hospital, Tokyo, JPN; 2 Department of Traditional Medicine, Toho University, Tokyo, JPN; 3 Department of Western Medicine, Hokushin-kai, Academic Society of Traditional Japanese Acupuncture and Moxibustion, Osaka, JPN; 4 Division of Palliative Care, Mitsui Memorial Hospital, Tokyo, JPN; 5 Department of Integrative/Complementary Medicine, Acupuncture Clinic, Seimei-in, Tokyo, JPN; 6 Department of Oriental Medicine, Hokushin-kai, Academic Society of Traditional Japanese Acupuncture and Moxibustion, Osaka, JPN

**Keywords:** acupoint exam, hokushin-kai style acupuncture, parkinson’s disease, pisa syndrome, traditional japanese acupuncture and moxibustion

## Abstract

Parkinson’s disease (PD) is a common progressive neurodegenerative disease. The management of PD including Pisa syndrome (PS), a postural deformity in PD characterized by reversible lateral bending of the trunk on the side, is often challenging. Recently, acupuncture has been a recognized intervention for treating motor or non-motor symptoms in PD management. However, very few of these studies or cases have been reported from Japan.

A 58-year-old man with a four-year history of PD (Hoehn and Yahr Scale: Stage 2) presented to the acupuncture department of our hospital with dysphasia, bradykinesia, forward posture, and newly appeared right-side bending of the trunk after he increased the dose of rotigotine delivered via skin patches six months earlier. There was no change in the right-sided bending of the trunk two months after the withdrawal of the dopaminergic agents. A traditional Japanese acupuncture and moxibustion treatment, *Hokushin-kai*, was started. According to the Oriental medical diagnosis, he was categorized with “liver depression,” “kidney deficiency,” and “dampness” patterns. The treatment was administered once a week, and only one or two needles were used. The acupoints, such as Ququan (LR8) or Houxi (SI3), were selected according to the Oriental medical diagnosis and the findings of the acupoint examination. At first, the Movement Disorder Society-Sponsored Revision of the Unified Parkinson’s Disease Rating Scale (MDS-UPDRS) score was 34 points, and the Parkinson’s Disease Questionnaire (PDQ-39) score was 42 points; the Cobb angle was 45°. After 10 weeks, he could walk smoothly and almost upright. MDS-UPDRS-3 and PDQ-39 scores improved to 12 points and 34 points, respectively, while the Cobb angle improved to 32°. Changes (improvements) in his gait and posture are shown in the videos included in this case report.

We present a case of PD bradykinesia, forward posture, and drug-induced PS alleviated with traditional Japanese acupuncture. This case report suggests that acupuncture using this Japanese method would achieve similar efficacies to those achieved in conventional case reports or clinical trials, and it could be one of the optional treatments available for PD. Further studies, such as the long-term effect of acupuncture on PD patients or improved outcomes of PD patients with early-phase intervention, are required.

## Introduction

Parkinson’s disease (PD) is a common, progressive neurodegenerative disorder predominantly affecting older adults, especially men. It is characterized by symptoms such as bradykinesia, muscular rigidity, rest tremor, and impairments in posture and gait. The incidence and prevalence of PD increase with age or exposure to certain environmental factors, such as neurotoxic agents [1].

Pisa syndrome (PS) is a postural deformity in PD defined as reversible lateral bending of the trunk with a tendency to lean to one side [2]. No consensus has been reached on the degree of lateral trunk flexion needed to define PS in PD; previous studies applied a cutoff of at least 10-15° of lateral flexion for the criteria of PS [2]. PS is also reported secondary to antipsychotic treatment, dementia, parkinsonism, and other neurodegenerative diseases or neurological disorders, including normal-pressure hydrocephalus and subdural hematoma. PS has been reported in PD patients after changes in dopaminergic treatment or as a complication of surgical procedures [2]. The prevalence of PS in PD patients varies in each investigation due to the absence of a consensus on PS diagnostic criteria or definitions.

The management of PD is often challenging. Pharmacotherapy, such as levodopa, could alleviate motor symptoms, although no medications have been shown to slow the progression of PD. Deep brain stimulation therapy is one choice for treating motor symptoms (except some symptoms such as impaired balance and freezing of gait); moreover, it is not effective in treating non-motor symptoms of PD (e.g., autonomic symptoms, cognitive impairment, mood changes, or apathy) and has a risk of stroke or infection [3]. Furthermore, PS is also clinically challenging because there is no evidence of effective treatment. PD patients with PS tend to fall more often and have a worse quality of life (QOL) compared to those without PS. PD drug revision and adjustment as well as other pharmacological, non-pharmacological, and surgical strategies are PS management options [2].

Acupuncture is a recognized treatment for PD management. Some clinical trials or meta-analyses suggest that acupuncture is effective for motor or non-motor symptoms in PD patients, although good-quality evidence is limited [4,5]. Recent randomized controlled trials (RCTs) have illustrated the efficacy of acupuncture in improving sleep quality, QOL, and anxiety in PD patients [6,7]. Good-quality clinical trials of acupuncture for treating the motor symptoms of PD are currently underway. However, almost all of these studies or cases were reported from China, with very few from Japan [8]. Here, we present a case in which bradykinesia, forward posture, and drug-induced PS in a PD patient were alleviated after intervention with *Hokushin-kai*-style acupuncture [9], a traditional Japanese acupuncture and moxibustion method.

## Case presentation

A 58-year-old man with a four-year history of PD presented to the acupuncture department in our hospital with dysphasia, bradykinesia, forward posture, and right-side bending of the trunk.

A dopamine transporter scan conducted four years before the presentation by a neurologist revealed a diagnosis of PD (Hoehn and Yahr Scale: Stage 2) and a finding of decreased dopamine receptor binding in the right striatum. Subsequently, he was treated with medication. Six months before the presentation, the patient increased the dosage of rotigotine (a once-daily transdermal patch treatment for PD) from one to two sheets. Consequently, his trunk bent to the right side and dopaminergic agents were stopped. As his right-side bending of the trunk still had no change two months after the withdrawal of dopaminergic agents, he elected to undergo acupuncture treatment. His motor symptoms were aggravated by fatigue, mental tension, coldness, and high humidity (during a rainy day).

**Figure 1:**
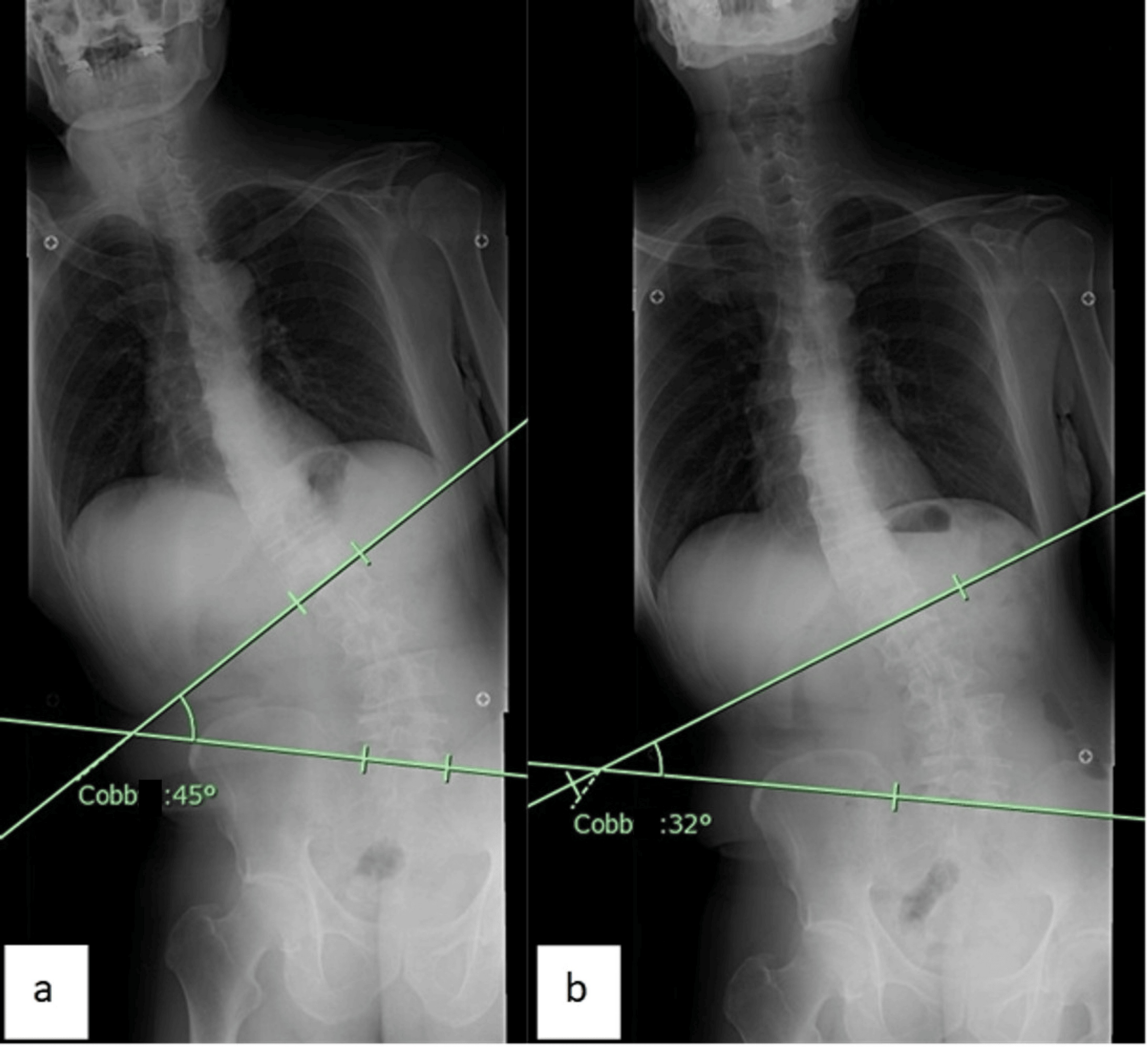
Spinal X-rays: changes from before to three months after acupuncture treatment. At first, the Cobb angle was 45° (a). Three months after the acupuncture intervention, the Cobb angle improved to 32° (b).

He had no past medical history apart from PD. His medications included levodopa, carbidopa, entacapone, rasagiline, trihexyphenidyl, istradefylline, zonisamide, and clonazepam. Upon examination, right-side bending of the trunk, facial masking, forward-leaning of the trunk, bradykinesia, dysphasia, micrographia, and muscular rigidity without rest tremors were noted. An acupoint examination revealed “deficiency” in left Zhaohai (KI6), bilateral Shenshu (BL23), and “excess” in right Neiguan (PC6), right Houxi (SI3), and left Taichong (LR3) (see below for details) (Figure 1).

According to the Oriental medical diagnosis (termed “pattern identification” based on the International Classification of Diseases 11th Revision), he was categorized as having a “liver depression,” “kidney deficiency,” and “dampness” pattern. We treated him accordingly with *Hokushin-kai*-style acupuncture, a traditional Japanese acupuncture and moxibustion method [9]. The treatment session occurred once a week. One or two sterilized disposable needles (Seirin Co., Shizuoka, Japan) were inserted into each acupoint and retained with no manipulations (depth of 4-10 mm at each acupoint). Acupoints, needle size, needling method, and retention time of each acupuncture treatment session are shown in Table 1. All of the needle insertions were performed 60-90 minutes after he took the PD medications. The acupoint for treatment was selected with the oriental medical diagnosis and the findings of the acupoint examination.

**Table 1:** Acupoints, needle size, needling method, and retention time of each acupuncture treatment session over 10 weeks. Acupuncture treatment was performed per week for a total of 11 sessions over 10 weeks. The number of treatments shows the details of each acupuncture treatment session. Only in the number 8 session, two needles were used for treatment. R: right; L: left; D: draining; T: tonifying method

Number of treatments	Acupoint	Needle size (diameter × length mm)	Needling method	Retention time (minutes)
1	L, Taichong (LR3)	0.2 × 15	D	15
2	L, Shenmai (BL62)	0.2 × 15	D	10
3	R, Ququan (LR8)	0.2 × 30	D	10
4	L, Weishu (BL21)	0.2 × 40	D	12
5	L, Zhangmen (LR13)	0.2 × 40	D	10
6	L, Ququan (LR8)	0.25 × 15	T	10
7	R, Houxi (SI3)	0.25 × 30	D	10
8-1	L, Baihui (GV20)	0.25 × 30	D	4
8-2	L, Zhaohai (KI6)	0.25 × 15	D	10
9	L, Ququan (LR8)	0.25 × 30	T	10
10	R, Houxi (SI3)	0.25 × 15	D	10
11	R, Fenglong (ST40)	0.2 × 40	D	10

After the procedure, his right-side bending of the trunk, forward-leaning of the trunk, bradykinesia, and muscular rigidity gradually improved. At first, the Movement Disorder Society-Sponsored Revision of the Unified Parkinson’s Disease Rating Scale (MDS-UPDRS-3), a scale of motor symptoms in PD [10], was 34 points and the Parkinson’s Disease Questionnaire (PDQ-39), a scale of QOL in PD [11], was 42 points. Although the PDQ-39 deteriorated to 61 points four weeks after starting acupuncture treatment, that period coincided with the rainy season in Japan. Ten weeks later, he could walk smoothly and almost upright. MDS-UPDRS-3 and PDQ-39 improved to 12 points and 34 points, respectively, while the Cobb angle improved to 32° (Figure 1).

**Figure 2:**
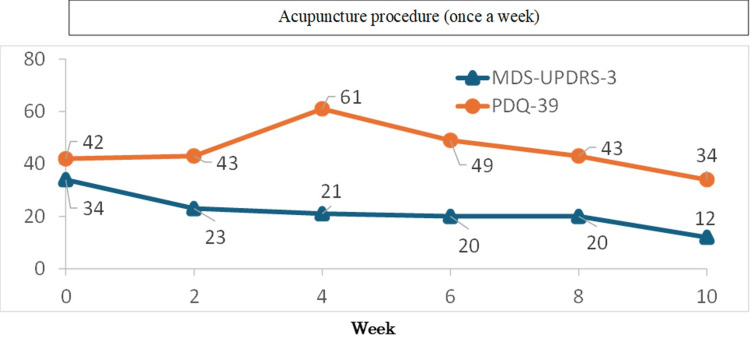
Therapeutic course of acupuncture treatment. At first, the MDS-UPDRS-3 score was 34 points, and the PDQ-39 score was 42 points. Though the PDQ-39 score deteriorated four weeks after he started acupuncture treatment, that period coincided with the rainy season in Japan as high humidity exacerbates the condition. Ten weeks later, the MDS-UPDRS-3 and PDQ-39 scores improved to 12 points and 34 points, respectively. MDS-UPDRS-3: Movement Disorder Society-Sponsored Revision of the Unified Parkinson’s Disease Rating Scale; PDQ-39: Parkinson’s Disease Questionnaire

As his dysphasia and micrographia remain, the acupuncture treatment continues. No adverse events have occurred, and acupuncture was performed by a clinical physician with two years of acupuncture experience. The therapeutic course of acupuncture treatment and changes (improvements) in his gait and posture are shown in Figure 2 and Videos 1-4, respectively (Video 1: before acupuncture treatment; Video 2: 10 minutes after the first acupuncture treatment; Video 3: one week after the first acupuncture treatment; Video 4: 11 weeks after the first acupuncture treatment).

**Video 1:** Changes (improvements) in the patient’s gait and posture before acupuncture treatment.

**Video 2:** Changes (improvements) in the patient’s gait and posture 10 minutes after the first acupuncture treatment.

**Video 3:** Changes (improvements) in the patient’s gait and posture one week after the first acupuncture treatment.

**Video 4:** Changes (improvements) in the patient’s gait and posture 11 weeks after the first acupuncture treatment.

## Discussion

This is the first case report of acupuncture treatment in a PD patient using the traditional Japanese *Hokushin-kai-*style method in English.

In a recent multicenter RCT from China, conventional pharmacological treatment combined with electroacupuncture significantly improved UPDRS-3 and PDQ-39 scores 12 weeks later [12]. Similarly, a single-center, prospective, observational, single-arm study from South Korea showed that the Meridian Activation Remedy System combined with exercise and acupuncture therapy significantly improved MDS-UPDRS-3 from 20.0 ± 11.8 to 8.8 ± 5.5 (p = 0.003) and PDQ-39 scores (not significantly) eight weeks later [13]. Moreover, a previous case report of electroacupuncture for PS in PD described how the UPDRS-3 score and Cobb angle improved four weeks later from 19 and 18.14° to 9 and 13.41°, respectively [14]. These results are similar to our case.

Acupuncture has long been known to relax excessive muscle tension by reducing the excitability activity of spinal motor neurons [15]. In addition, a study outlining the mechanism of acupuncture for PD revealed that acupuncture may protect dopaminergic neurons from degeneration via antioxidative stress, anti-inflammatory, and antiapoptotic pathways, as well as modulating the neurotransmitter balance in the basal ganglia circuit [16]. Recent research for PD patients via functional magnetic resonance imaging revealed that acupuncture may alleviate motor symptoms by modulating the cerebello-thalamo-cortical circuit [17]. Interestingly, acupuncture is considered to have a synergistic effect with levodopa and other anti-PD drugs, just like in our case.

*Hokushin-kai*-style acupuncture [9] is a traditional form of Japanese acupuncture and moxibustion that we used in this case. One of its characteristics is that only one or two needles are used for each treatment session without manipulation or electricity compared to the 10-20 needles needed with or without manipulation or electricity for other methods. In the *Hokushin-kai* style, taking the detailed history of the patient and performing a detailed acupoint examination are regarded as very important in selecting the acupoint for treatment. Recently, the concept of “acupoint sensitization” has been proposed [18]. There is sensory hypersensitivity and functional plasticity in sensitized acupoints, and a better clinical effect would be given for selecting such acupoints for treatment. It is speculated that sensitized acupoints are well-selected with the acupuncture practitioner of the *Hokushin-kai* style. In this case, only one needle could succeed in harmonizing the uneven distribution of muscle tone throughout the whole body in this patient.

**Figure 3:**
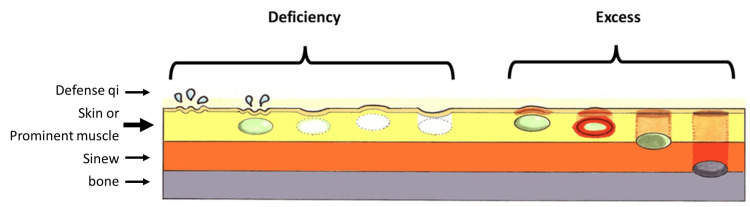
Classification of the findings in the acupoint examination. In Oriental medicine, defense qi protects the body such as the skin, limb joint, and eye outside the meridian from pathogens (e.g., infection, rapid climate change). Image credits: Yu Takeshita.

Acupoint examination findings are classified into two categories, namely, deficiency and excess (Figure 3). In the state of deficiency, perspiration or coldness on the surface of the skin and flaccidity on the skin surface or in the deep subcutaneous area are felt. In the state of excess, heat, tension, induration, and tenderness appear on the skin surface or in the deep subcutaneous areas. These findings change according to the physical and mental state of the patient, the weather, or the type of acupuncture treatment. However, these findings are sometimes quite vague and difficult for acupuncture practitioners to recognize. Therefore, several years of training are required to recognize acupoint findings more accurately [9].

This case has the following limitations. First, as the follow-up period for the patient was only 10 weeks, the long-term clinical effects are unknown. Second, his motor symptoms were improved only during “on-time,” and he could not move satisfactorily in “off-time” the same as before the acupuncture treatment. This might have been due to the inability to treat with acupuncture satisfactorily due to his progressive phase of PD and extensive depletion of dopamine neurons in the basal ganglia.

Recent investigations showed that physical activity improved all-cause mortality in PD patients [19]. Therefore, acupuncture could help them exercise or prevent them from becoming bedridden by relaxing their muscles as well as reducing the burden on caregivers, which is another problem associated with PD [20].

Further studies, such as the long-term effect of acupuncture on PD patients, improving outcomes of PD patients with early-phase intervention, or a combination of acupuncture with other non-invasive treatments (e.g., repetitive transcranial magnetic stimulation [21]) for motor symptoms are required.

## Conclusions

We present a case outlining how PD bradykinesia, forward posture, and drug-induced PS were alleviated with traditional Japanese acupuncture. This case suggests that acupuncture using this traditional Japanese method has a similar clinical effect, as shown in previous case reports or clinical trials, and would be an optional treatment for PD. Identifying the sensitized acupoints with an acupoint examination would be important to show more excellent clinical effects with one-needle acupuncture. Further studies, such as the long-term effects of acupuncture for PD patients or improving the outcomes of PD patients with early-phase intervention, are required.
